# Upregulation of Atypical Cadherin FAT1 Promotes an Immunosuppressive Tumor Microenvironment *via* TGF-β

**DOI:** 10.3389/fimmu.2022.813888

**Published:** 2022-05-26

**Authors:** Khushboo Irshad, Chitrangda Srivastava, Nargis Malik, Manvi Arora, Yakhlesh Gupta, Sanjeev Goswami, Chitra Sarkar, Vaishali Suri, Swati Mahajan, Deepak Kumar Gupta, Ashish Suri, Parthaprasad Chattopadhyay, Subrata Sinha, Kunzang Chosdol

**Affiliations:** ^1^ Department of Biochemistry, All India Institute of Medical Sciences, New Delhi, India; ^2^ Department of Pathology, All India Institute of Medical Sciences, New Delhi, India; ^3^ Department of Neurosurgery, All India Institute of Medical Sciences, New Delhi, India

**Keywords:** FAT1, immunosuppression, cancer, transforming growth factor (TGF), inflammation

## Abstract

FAT atypical cadherin 1 (FAT1) promotes glioblastoma (GBM) by promoting protumorigenic inflammatory cytokine expression in tumor cells. However, tumors also have an immunosuppressive microenvironment maintained by mediators such as transforming growth factor (TGF)-β cytokines. Here, we have studied the role of FAT1 in tumor immune suppression. Our preliminary TIMER2.0 analysis of The Cancer Genome Atlas (TCGA) database revealed an inverse correlation of FAT1 expression with infiltration of tumor-inhibiting immune cells (such as monocytes and T cells) and a positive correlation with tumor-promoting immune cells [such as myeloid-derived suppressor cells (MDSCs)] in various cancers. We have analyzed the role of FAT1 in modulating the expression of TGF-β1/2 in resected human gliomas, primary glioma cultures, and other cancer cell lines (U87MG, HepG2, Panc-1, and HeLa). Positive correlations of gene expression of FAT1 and TGF-β1/2 were observed in various cancers in TCGA, Glioma Longitudinal Analysis Consortium (GLASS), and Chinese Glioma Genome Atlas (CGGA) databases. Positive expression correlations of FAT1 were also found with TGF-β1/2 and Serpine1 (downstream target) in fresh-frozen GBM samples using q-PCR. siRNA-mediated *FAT1* knockdown in cancer cell lines and in primary cultures led to decreased TGF-β1/2 expression/secretion as assessed by q-PCR, Western blotting, and ELISA. There was increased chemotaxis (transmigration) of THP-1 monocytes toward siFAT1-transfected tumor cell supernatant as a consequence of decreased TGF-β1/2 secretion. Reduced TGF-β1 expression was also observed in THP-1 cultured in conditioned media from FAT1-depleted glioma cells, thus contributing to immune suppression. In U87MG cells, decreased TGF-β1 upon *FAT1* knockdown was mediated by miR-663a, a known modulator. FAT1 expression was also observed to correlate positively with the expression of surrogate markers of MDSCs [programmed death ligand-1 (PD-L1), PD-L2, and interleukin (IL)-10] in glioma tumors, suggesting a potential role of FAT1 in MDSC-mediated immunosuppression. Hence, our findings elaborate contributions of FAT1 to immune evasion, where FAT1 enables an immunosuppressive microenvironment in GBM and other cancers *via* TGF-β1/2.

## Introduction

FAT atypical cadherin 1 (FAT1) is expressed as a transmembrane protein and has been investigated only in recent years for its role in human cancers. The role of FAT1 has been attributed as context-dependent and tissue-specific in different cancers (1–4). While some forms of malignancies such as skin squamous cell carcinoma, lung cancer, head and neck squamous cell carcinoma, and oral cancer have been associated with mutations and deletion in the *FAT1* gene (4–6), other cancers including breast carcinoma, colorectal cancer, hepatocellular carcinoma, cervical cancer, pancreatic cancer, and gliomas (3, 7–13) have shown an oncogenic role of FAT1 in tumor pathogenesis. However, as a relatively new gene implicated in cancer, it remains to be explored how FAT1 aberrations might contribute to various hallmarks of cancer (14) leading to tumor development and progression. We have earlier shown the role of FAT1 in promoting epithelial–mesenchymal transition (EMT), invasiveness, hypoxia-activated signaling, stemness, and clonogenicity in glioblastoma (GBM) cells (11–13). FAT1 was found to promote the expression of pro-inflammatory mediators such as interleukin (IL)-6, cyclooxygenase (COX)-2, IL-1β, and vascular endothelial growth factor (VEGF)-C in a homogeneous microenvironment of glioma cells where they are likely to sustain glioma growth by serving as autocrine prosurvival cytokines (11, 15–17). However, an emerging hallmark of cancer describes evasion of immune response as a critical characteristic of most solid tumors (14), including glioma (18, 19).

The most well-known and potent immunosuppressive mediators that aggravate tumor progression are transforming growth factor (TGF)-β cytokines (20–22). TGF-β cytokines are crucial mediators of immune homeostasis that inhibit expansion and functions of different immune cell types such as effector T cells, macrophages, natural killer (NK) cells, and antigen-presenting dendritic cells (DCs) (21, 23). The immunosuppressive effects of TGF-β enabling cancer progression and development of TGF-β inhibitors as antitumor agents have been discussed in the context of various cancers such as liver, cervical, breast, colon, lung, prostate, esophageal, and pancreatic cancer (23, 24). Particularly, in GBM, elevated TGF-β levels have often been associated with the immunosuppressed status of patients and, therefore, as responsible for loss of tumor immune surveillance (25). Thus, keeping in mind the complexity of the GBM microenvironment, we sought to check for any potential role of FAT1 in regulating the expression of TGF-β cytokines, especially in the context of heterotypic cell interactions.

Another important component of the intratumoral immunosuppressive microenvironment that has been associated with clinical cancer stage and diminished response to therapy is increased infiltration of myeloid-derived suppressor cells (MDSCs) (26). These cells are characterized by the production of IL-10 immunosuppressive cytokine and upregulation of immunoregulatory mediators programmed death ligand-1 (PD-L1)/PD-L2 (27–30), which are being actively evaluated as targets for cancer immunotherapy (31).

In this study, we have investigated the functional role of FAT1 in the regulation of TGF-β expression and production in tumor cells. We have assessed the effect of modulated TGF-β secretion from FAT1-depleted tumor cells on *in vitro* migration and phenotype of monocyte-derived cell line. Our results indicate a potential role of FAT1 in promoting an immunosuppressive microenvironment in gliomas, which might extend to other cancers as well.

## Materials and Methods

### TIMER2.0 Database Analysis

TIMER2.0 web server (Tumor IMmune Estimation Resource; http://timer.cistrome.org) (32) was used to visualize the correlation between the *FAT1* gene expression and the level of immune infiltration found in cases of different cancers from The Cancer Genome Atlas (TCGA) database. The current release of TIMER has incorporated 10,009 samples across 23 cancer types from TCGA. Gene module was selected under “immune association,” and *FAT1* was selected as the gene of interest. Types of immune cells were selected under “immune infiltrates.” “Purity adjustment” option was selected for the use of partial Spearman’s correlation in the association analysis that was presented in the form of a heat map.

### *In Silico* Expression Analysis of FAT1 and Immunosuppressive Genes in Different Cancers

Normalized mRNA expression data for FAT1, TGF-β1, TGF-β2, and Serpine1 genes were obtained for 572 GBM cases from the public database of TCGA (https://www.cancer.gov/tcga). Gene expression data of 102 glioma cases were retrieved from the public database named Glioma Longitudinal Analysis Consortium (GLASS; www.synapse.org/glass) (33). TCGA gene expression data for pancreatic, liver, cervical, and colorectal cancers were obtained from http://www.proteinatlas.org where fragments per kilobase per million (FPKM) values are provided from RNA-Seq analyses (data not normalized with respect to normal tissues). For gene expression correlation analysis in the Chinese Glioma Genome Atlas (CGGA; www.cgga.org.cn), glioma cases from mRNA_array_301 dataset and mRNAseq_693 dataset were used. Gene-to-gene correlation analysis was performed using Spearman’s test in SPSS v20.0 or GraphPad Prism v5.00 statistical software.

### Patient Samples and Ethics Statement

Fifty glioma tumors (49 GBM and one grade II glioma) were obtained from the Department of Neurosurgery/Neuropathology, AIIMS, New Delhi, after obtaining approval from the Institute Ethics Committee (Ref no. IECPG-3/28.10.2015, RT-16/30.12.2015, IECPG/184/2/2019) and due patient consent as either fresh-frozen or freshly resected samples. Normal human brain total RNA was purchased commercially (Clontech) for use as control sample in q-PCR analysis.

### Generation of Primary Cultures of Human Glioma Cells From Surgical Tumors

Primary cell cultures (details in **Table S1**) were generated from freshly resected human glioma tumors (grades II and IV) using mechanical mincing/Accutase^®^ treatment and maintenance in Dulbecco's Modified Eagle Medium (DMEM) F-12 + 10% fetal bovine serum (FBS). These tumors were collected immediately from the Neurosurgery operation theater, and the histopathological diagnosis/grading was obtained later from the Department of Neuropathology, AIIMS.

### Culture of Cancer Cell Lines

U87MG was used as a representative GBM cell line in most of the experiments. U87MG, along with other cancer cell lines such as HeLa (cervical cancer), Panc-1 (pancreatic cancer), and HepG2 (hepatocellular cancer), was maintained in DMEM + 10% FBS + 10 μg/ml ciprofloxacin in 25-cm^2^ culture flasks in Anoxomat chamber.

### siRNA Transfection in Cell Cultures

Primary glioma cultures (n = 2; namely, PC-A: grade II oligodendroglioma; and PC-B: grade IV GBM) and other cancer cell lines (U87MG, HeLa, HepG2, and Panc-1) were transfected with siFAT1 (FAT1 Stealth siRNA #HSS103567, Invitrogen) and siControl (Stealth RNAi™ siRNA Negative Control Med GC Duplex #3 Cat #12935113) using Lipofectamine 3000 (Invitrogen). After 72 h of siRNA treatment, culture supernatants were harvested, and total RNA was isolated from the transfected cells using Tri reagent (Sigma-Aldrich). *FAT1* gene knockdown was confirmed by q-PCR (Corbett, Qiagen) using β-actin as an internal control reference.

### Gene Expression Analysis in Fresh-Frozen Glioblastoma Tumors and Cell Culture Samples

Expression levels of FAT1 and genes associated with immunosuppressive pathways/molecules (TGF-β1, TGF-β2, Serpine1, PD-L1, PD-L2, and IL-10) were analyzed using q-PCR (Corbett, Qiagen) with respect to 18S rRNA internal control reference in RNA samples extracted from fresh-frozen GBM tumors (n = 49). The same genes were also checked for modulation in response to siRNA-mediated transient *FAT1* knockdown (72 h) in cancer cell lines and primary cultures with respect to 18S rRNA or β-actin. Gene-specific primers were designed using Primer3 and synthesized commercially. The primer sequences are listed in **Table S2**. Primers were standardized for their annealing temperature and optimum PCR conditions. Fold expression ratios obtained from ΔΔCt analysis were subjected to Spearman’s correlation analysis using SPSS v20.0 and GraphPad Prism v5.00. Cluster 3.0 and TreeView software was used for gene clustering analyses.

### miRNA Analysis

For analysis of miRNA, total RNA was isolated using miRNeasy kit (Qiagen; cat #217004). For the quantification of target miRNA, first-strand synthesis was done with 1 µg RNA using miRCURY LNA™ RT kit (Qiagen; cat #339340). Predesigned q-PCR primers for miR-663a and U6 were purchased from Exiqon (Qiagen). Expression of miR-663a was normalized to that of the internal control reference U6. Fold expression ratios were calculated by ΔΔCt analysis. Inhibition of miR-663a was done using Anti-miR™ miRNA inhibitor oligonucleotide (Thermo Fisher Scientific, USA; cat #AM17000) that was used to transfect U87MG cells using Lipofectamine 3000 (Invitrogen). mirVana™ miRNA mimic Negative Control #1 (Thermo Fisher Scientific, USA; cat #4464058) was used as the control oligonucleotide in transfections. RNA was isolated from these cells at 48 h of transfection.

### Western Blot Analysis

Whole-cell lysates were prepared from siFAT1/siControl-transfected cells at 72 h using RIPA buffer (Thermo Fisher Scientific), protease inhibitor cocktail (Sigma-Aldrich), and phosphatase inhibitor cocktail (Sigma-Aldrich). In this study, 40–100 µg of lysates were loaded in 5%–15% sodium dodecyl-sulfate polyacrylamide gel electrophoresis (SDS-PAGE) gels and electrophoresed in Western blot apparatus (Bio-Rad). Primary antibodies used were as follows: TGF-β1 (rabbit polyclonal, 1:1,000, Abcam, #ab92486), TGF-β2 (mouse monoclonal, 1:1,000, Abcam, #ab36495), Serpine1/PAI-1 (rabbit polyclonal, 1:1,000, Abcam, #ab66705), SMAD2/3 (rabbit monoclonal, 1:1,000, Cell Signaling Technology, #8685), and β-actin (mouse monoclonal, 1:5,000, Abbkine).

### Multi-Analyte ELISArray for Detection of Released Cytokines

Culture supernatants from siFAT1/siControl-transfected cells (PC-A, PC-B, U87MG, HeLa, and HepG2 cells) were harvested at 72 h post siRNA transfection. The collected supernatants were centrifuged at 1,500 rpm for 10 min and stored at –80°C until use. For analysis, the samples were thawed on ice and added to the human Th1/Th2/Th17 cytokines Multi-Analyte ELISArray plate (Qiagen). Absorbance readings of anti-inflammatory cytokines (IL-10, TGF-β1, IL-13, IL-4, IL-5) and pro-inflammatory cytokines [IL-2, IL-6, IL-12, IL-17A, interferon (IFN)-γ, tumor necrosis factor (TNF)-α, granulocyte colony-stimulating factor (G-CSF)] were taken at 450 nm in the same ELISA plate. Negative and positive controls were supplied in the kit and used as recommended. Relative levels of cytokines were determined as per manufacturer’s user manual.

### Culture of THP-1 Cell Line

We have used THP-1, a monocyte-derived cell line, as a representative of the monocyte lineage to validate key observations (34). THP-1 cells were maintained as a suspension culture in RPMI-1640 + 10% FBS + 1% pen-strep in 25-cm^2^ culture flasks in Anoxomat chamber. For monocyte polarization assessment, THP-1 monocytes were cultured for 24 h in conditioned media (culture supernatants) from siFAT1/siControl-transfected U87MG/PC-A/PC-B cells. This was followed by total RNA isolation from THP-1 cells, cDNA synthesis, and q-PCR for TGF-β1 gene with respect to β-actin gene.

### THP-1 Monocyte Migration Assay

To analyze the transmigration behavior of THP-1 monocytes toward conditioned media harvested from siFAT1/siControl-treated cells (U87MG, PC-A, PC-B, and HeLa), we used 24-transwell inserts (Corning; pore size 8 μm). Here, 3 × 10^5^ THP-1 cells in 200 μl serum-free RPMI-1640 was loaded on the upper chamber of the insert. Also, 500 μl culture supernatant of the siFAT1/siControl-transfected cells (U87MG, PC-A, PC-B, or HeLa) was added to the lower chamber. THP-1 monocytes were allowed to migrate for 16 h under standard culture conditions. THP-1 cells that migrated across the insert membrane to the lower chamber were photographed microscopically. Transmigrated THP-1 cells in each well were counted in at least five non-overlapping microscopic fields using ImageJ software and presented as average ± SD. The difference between THP-1 migration toward siFAT1/siControl media was analyzed using Student’s *t*-test, and p < 0.05 was considered statistically significant. Experiments were repeated thrice.

## Results

### FAT1 Expression Correlates Inversely With Immune Cell Infiltration Level in Different TCGA Cancers Including Glioblastoma Tumors

In order to investigate a possible role of FAT1 in tumor-associated immunosuppression, we checked for any correlation between the expression of FAT1 in tumors and levels of immune cell infiltration using TIMER2.0. TIMER2.0 is a comprehensive resource for analysis of immune infiltrates across diverse cancer types from TCGA database and provides visualization of the association between immune infiltrates and gene expression. As seen in **Figures S1A–C**, upon selecting for FAT1 gene expression, an inverse correlation was observed with infiltration levels of monocytes, CD8+ T cells, CD4+ T cells, NK cells, and dendritic cells in TCGA GBM cases (n = 153). On the contrary, infiltration levels of MDSCs and regulatory T cells (T-reg) cells, which are known to display immunosuppressive activity in tumors (35), were seen to correlate positively with FAT1 expression in TCGA GBM tumors (**Figures S1A–C**). However, among macrophages, FAT1 expression correlated positively with M0 macrophage infiltration; while it correlated inversely with the infiltration of both M1 and M2 macrophages, which entails further study in detail.

We also observed in cases of cervical cancer [cervical and endocervical cancer (CESC); n = 306] that there is mostly an inverse correlation of FAT1 expression with infiltration levels of CD8+ T cells, CD4+ T cells, and dendritic cells and a positive correlation with infiltration of MDSCs (**Figures S1A–C**). Similarly, in pancreatic cancer cases [pancreatic adenocarcinoma (PAAD); n = 179], FAT1 expression correlated inversely with infiltration of monocytes, CD8+ T cells, CD4+ T cells, and dendritic cells, while it correlated positively with that of MDSCs. Also, in liver cancer cases [liver hepatocellular carcinoma (LIHC); n = 371], FAT1 expression correlated inversely with infiltration of CD8+ T cells and dendritic cells while correlating positively with T-reg cell infiltration (**Figures S1A–C**). We thus concluded that FAT1 expression level in various cancers is inversely associated with infiltration of tumor-inhibiting immune cells and positively associated with the level of tumor-promoting immune cells.

### FAT1 Expression Correlates Positively With the Expression of Anti-Inflammatory Mediators TGF-β1 and TGF-β2 in Glioblastoma and Other Cancers

In order to find a probable link between FAT1 expression in tumor samples and immune cell infiltration levels as indicated by TIMER2.0 results, we checked for any association between the expression of FAT1 and that of known immunosuppressive cytokines, TGF-β1 and TGF-β2.

We analyzed the mRNA expression of FAT1 along with TGF-β1 and TGF-β2 in a tumor dataset containing 49 fresh-frozen human GBM tissues collected at AIIMS, New Delhi. We also included Serpine1 plasminogen activator inhibitor 1 (PAI-1), a well-known TGF-β downstream target gene (36–39), as a readout marker of modulated TGF-β1 and TGF-β2. The genes were analyzed by q-PCR with respect to 18S rRNA internal control reference and normalized by the levels found in normal human brain RNA (Clontech, Takara Bio, USA). Fold expression ratios were calculated using ΔΔCt method (**Table S3**) and subjected to Spearman’s correlation analysis (**Table S4**). We found significant positive correlation coefficients between the expression of FAT1 and TGF-β1 (r = 0.517; p ≤ 0.01), TGF-β2 (r = 0.523; p ≤ 0.01), and Serpine1 (r = 0.512; p ≤ 0.01) (**Table S4**), indicating a positive association between upregulated FAT1 expression (>2-fold in 44/49 GBM tumors; **Table S3**) and increased TGF-β1, TGF-β2, and Serpine1 in the studied GBM tumor samples.

**Figure 1:**
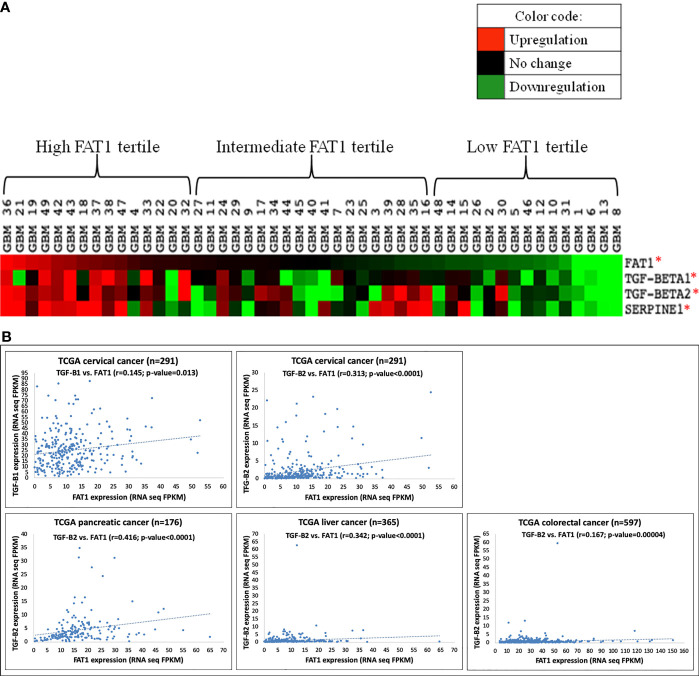
**(A)** Heat map showing semisupervised gene clustering of TGF-β1, TGF-β2, and Serpine1 expression in 49 GBM tumors arranged in decreasing order of FAT1 expression. Gene expression values found significantly different across high (n = 15) and low (n = 15) FAT1 tertiles have been marked with an asterisk (*) indicating p-value ≤0.05. **(B)** Scatter plots showing a positive correlation of FAT1 expression with the expression of TGF-β1 and TGF-β2 in various types of cancers (cervical, pancreatic, liver, and colorectal cancers) as analyzed in TCGA cases. TCGA, The Cancer Genome Atlas; FPKM, fragments per kilobase per million.

Next, we arranged the studied GBM tumors in decreasing order of FAT1 expression and grouped them by tertiles as follows: high (≥8.59-fold; 15 tumors); intermediate (3.74- to 7.83-fold; 19 tumors), and low (≤3.59-fold; 15 tumors) FAT1 GBM tertiles (**Table S5**). We generated a heat map to visualize semisupervised gene clustering in 49 tumors ordered in decreasing order of FAT1 expression by using Cluster 3.0 and TreeView software (**Figure 1A**). TGF-β1, TGF-β2, and Serpine1 displayed greater upregulation in the high FAT1 GBM tertile as compared to low FAT1 GBM tertile, and these expression values were found to be significantly different across the high and low tertiles (p ≤ 0.05) as analyzed using Student’s *t*-test (**Figure 1A**, **Table S5**).

For further validation of the above findings, gene expression correlation analysis was performed in the GBM dataset (n = 572) downloaded from TCGA website, where we found a significant positive correlation of the expression of FAT1 with TGF-β1 (r = 0.119, p = 0.002) and TGF-β2 (r = 0.123, p = 0.002) (**Table S6**). Similarly, expression correlation analysis in CGGA database yielded a positive correlation of FAT1 with TGF-β1 in primary glioma cases (r = 0.503, p < 0.01) and recurrent glioma cases (r = 0.865, p = 0.05) in mRNA_array_301 dataset (**Figure S2A**). In mRNAseq_693 dataset of CGGA, FAT1 expression was observed to be positively correlated with TGF-β2 expression in primary glioma cases (r = 0.344, p < 0.01) and recurrent glioma cases (r = 0.447, p < 0.01) (**Figure S2B**). We also checked GLASS that is a new public gene expression database of glioma samples having 90% non-TCGA cases (33). TGF-β1 was observed to be significantly correlated with FAT1 expression in GLASS glioma tumors (n = 102; r = 0.352, p = 0.0001) (**Table S7**).

Moreover, the expression of TGF-β1 (r = 0.145, p = 0.013) and TGF-β2 (r = 0.313, p = 4.910e-008) was also observed to be correlated with FAT1 expression in TCGA cervical cancer cases obtained from www.proteinatlas.org (**Figure 1B**). Similarly, TGF-β2 was observed to be correlated with FAT1 in pancreatic cancer cases (r = 0.416, p = 9.61e-009), liver cancer cases (r = 0.342, p = 2.00e-011), and colorectal cancer cases (r = 0.167, p = 0.00004) of TCGA database (**Figure 1B**). Hence, the positive correlation between the expression of FAT1 and TGF-β1/TGF-β2 extends to other cancers as well, in addition to GBM tumors.

Hence, the above findings confirmed a positive association between the expression of FAT1 and the expression of immunosuppressive mediators TGF-β1 and TGF-β2 in cancers including glioma.

### *FAT1* Knockdown Leads to Decreased Expression and Secretion of Immunosuppressive Mediators by Glioma and Other Cancer Cells

**Figure 2:**
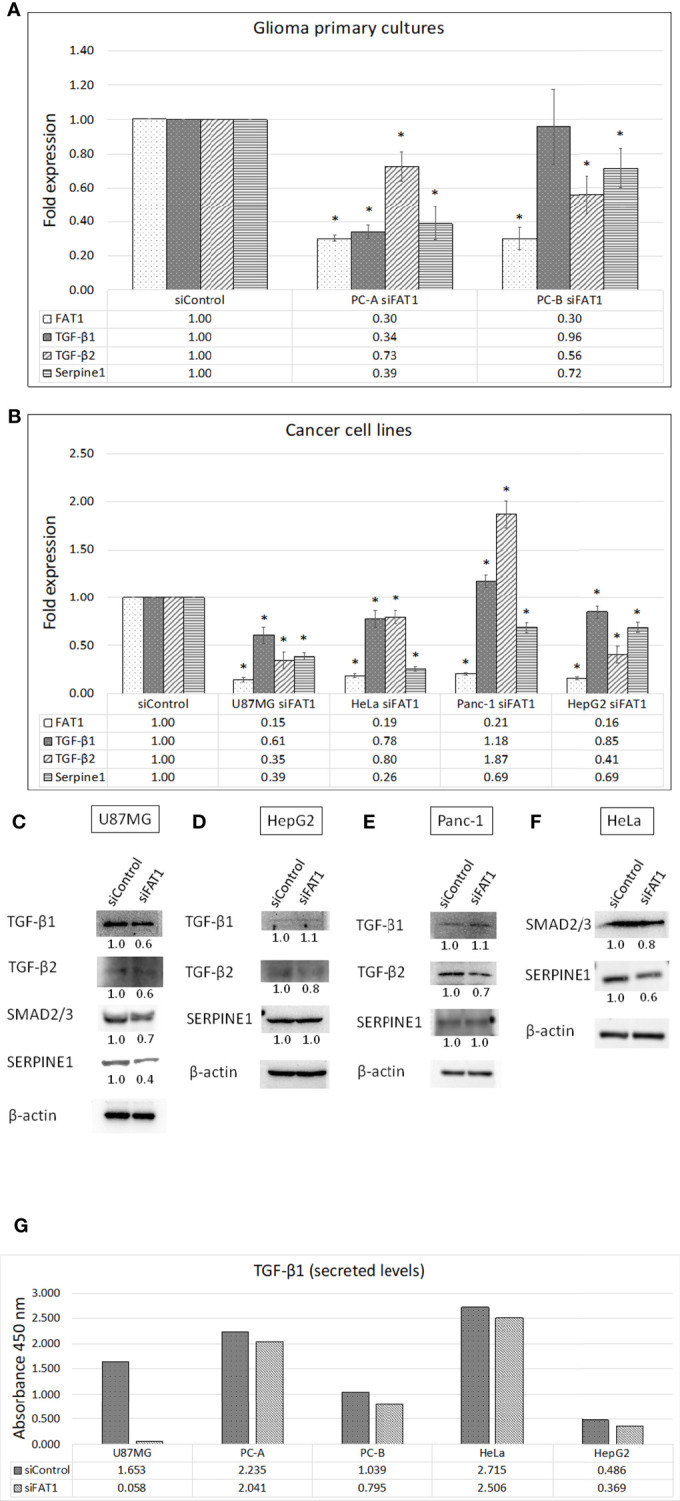
Effect of *FAT1* knockdown on the expression of TGF-β1, TGF-β2, and Serpine1 genes in **(A)** glioma primary cultures (PC-A: grade II oligodendroglioma; and PC-B: GBM) and **(B)** representative cancer cell lines (U87MG, HeLa, Panc-1, and HepG2) as assessed by q-PCR. Relative gene expression in each siFAT1 sample has been normalized to its respective siControl sample. (*) indicates p-value ≤0.05. **(C–F)** Western blots showing the expression of TGF-β1/2 pathway molecules (TGF-β1, TGF-β2, SMAD2/3 dimer, SERPINE1/PAI-1) in whole-cell lysates of siFAT1 cells vs. siControl cells (U87MG, HepG2, Panc-1, and HeLa) at 72 h of *FAT1* knockdown. In this study, β-actin was used as the loading control. Densitometric values of the proteins analyzed with respect to β-actin have been provided below the blots. **(G)** Graph showing optical densities of TGF-β1 cytokine secreted by siControl cells vs. siFAT1 cells in U87MG, PC-A, PC-B, HeLa, and HepG2 culture supernatants depicting the modulation of cytokine release by tumor cells upon *FAT1* knockdown.

In order to check for the functional role of FAT1 in the modulation of immunosuppressive mediators in cancer cells, we generated short-term primary cultures from human glioma tumors (PC-A: grade II oligodendroglioma; and PC-B: grade IV GBM) (**Table S1** and **Figure 2A**) and analyzed the effect of siRNA-mediated transient knockdown of *FAT1* gene on the expression of TGF-β1 and TGF-β2 and their downstream target, Serpine1. We achieved successful *FAT1* knockdown (70% knockdown) at 72 h of siFAT1 transfection in both primary cultures (**Figure 2A**). While TGF-β1 mRNA level was found to be significantly downregulated in PC-A upon FAT1 depletion, TGF-β2 mRNA was seen to be decreased in both PC-A and PC-B (**Figure 2A**). Serpine1 was also reduced in both PC-A and PC-B after *FAT1* knockdown (**Figure 2A**). Thus, FAT1 depletion in patient-derived glioma cells results in a downregulated mRNA expression of TGF-β1 and TGF-β2, which is reflected by decreased Serpine1 expression as well.

We also checked for modulation of the TGF-β1/2 expression in response to *FAT1* knockdown in representative cell lines of glioma and other cancers such as U87MG (GBM), HeLa (cervical cancer), Panc-1 (pancreatic cancer), and HepG2 (hepatocellular cancer). In this study, ≥79% *FAT1* knockdown was achieved in all cell lines at 72 h of siFAT1 transfection (**Figure 2B**). We observed decreased TGF-β1 and TGF-β2 in siFAT1-treated U87MG cells as compared to siControl-treated cells at both mRNA and protein levels (**Figures 2B, C**). As a result, SMAD2/3 (dimer) and Serpine1 (PAI-1) that are both TGF-β pathway effector molecules were also found to be decreased at the mRNA and protein levels in response to *FAT1* knockdown in U87MG (**Figures 2B, C**).

In HeLa cells, mRNA levels of TGF-β1 and TGF-β2 were downregulated by 20%–22% upon *FAT1* knockdown; while in HepG2 cells, TGF-β1 and TGF-β2 levels were downregulated by 15% and 59%, respectively (**Figure 2B**). At the protein level, reduced TGF-β2 was found in FAT1-depleted HepG2 and Panc-1 cells (although TGF-β1 mRNA was slightly increased) (**Figures 2D, E**). HeLa cells also displayed a reduction in SMAD2/3 and Serpine1 protein levels upon *FAT1* knockdown (**Figure 2F**). Serpine1 protein did not alter much in HepG2 and Panc-1 cells, but Serpine1 mRNA was observed to be decreased in all cell lines after *FAT1* knockdown (**Figures 2B, D, E**).

Next, we studied the effect of *FAT1* knockdown on the secretion of cytokines from tumor cells using multi-analyte ELISArray kit (Qiagen). Toward this, supernatants from siControl- and siFAT1-treated cultures were collected at 72 h post-transfection in glioma cells (U87MG and primary cultures PC-A and PC-B), HeLa cells, and HepG2 cells (**Figure 2G**). Among anti-inflammatory cytokines, only TGF-β1 showed detectable absorbance signal at 450 nm (color development). Relative to siControl cells, secreted TGF-β1 level was observed to be reduced in culture supernatants of siFAT1-treated U87MG, PC-A, PC-B, HeLa, and HepG2 (**Figure 2G**). Thus, we found a relative decrease in TGF-β1 secretion from FAT1-depleted tumor primary cultures and other studied cancer cell lines. Therefore, we concluded that FAT1 regulates the secretion and/or expression of TGF-β1 and TGF-β2, which are well-known immunosuppressive cytokines, in glioma and other cancer cells.

In addition, in the multi-analyte ELISArray results, we also noted a decrease in IL-6 (pro-inflammatory cytokine) secretion in culture supernatants of siFAT1-transfected U87MG and PC-B cells as compared to respective siControl cells (**Figure S3**). Thus, FAT1 regulates the release of both anti-inflammatory and pro-inflammatory cytokines from GBM cells.

### FAT1-Mediated Modulation of Immunosuppressive Cytokine Secretion Leads to Altered Migration of THP-1 Monocytes Toward Supernatants of FAT1-Attenuated Tumor Cells

Since we found that *FAT1* knockdown in tumor cells leads to reduced expression and secretion of TGF-β1/TGF-β2 immunosuppressive cytokines, we next checked if this results in altered migration of monocytes in response to conditioned media from siFAT1-transfected tumor cells as compared to media from siControl-transfected tumor cells. For this, an *in vitro* THP-1 monocyte migration model (34) was used where THP-1 cells were allowed to migrate across 8-μm pore-size Transwell culture inserts for 16 h toward conditioned media from siFAT1/siControl-transfected tumor cells (U87MG, PC-A, PC-B, and HeLa).

**Figure 3:**
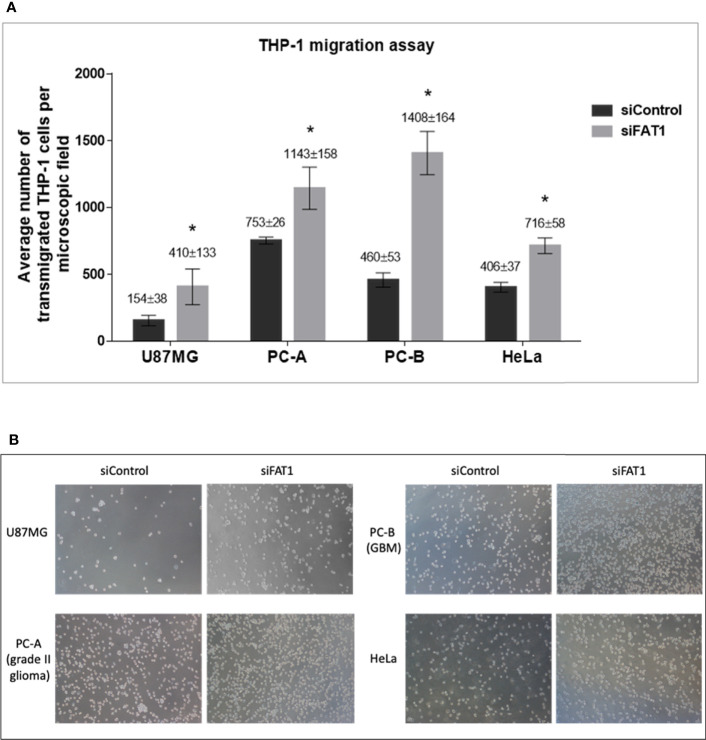
**(A)** Graphs showing the average number of transmigrated THP-1 cells per microscopic field in response to conditioned medium (culture supernatants) from siControl/siFAT1-transfected U87MG, PC-A, PC-B, and HeLa cells, depicting the modulation of monocyte migration in response to *FAT1* knockdown in tumor cells. Transmigrated THP-1 cells have been counted in five non-overlapping microscopic fields using ImageJ software, and the number has been presented as average ± SD. Significance has been indicated by (*) as p-value ≤0.01. Experiments were repeated thrice. **(B)** Microscopic images (10× magnification) of transmigrated THP-1 cells in response to conditioned media (supernatants) from siControl/siFAT1-transfected tumor cells, i.e., U87MG, PC-A, PC-B, and HeLa.

**Figure 4:**
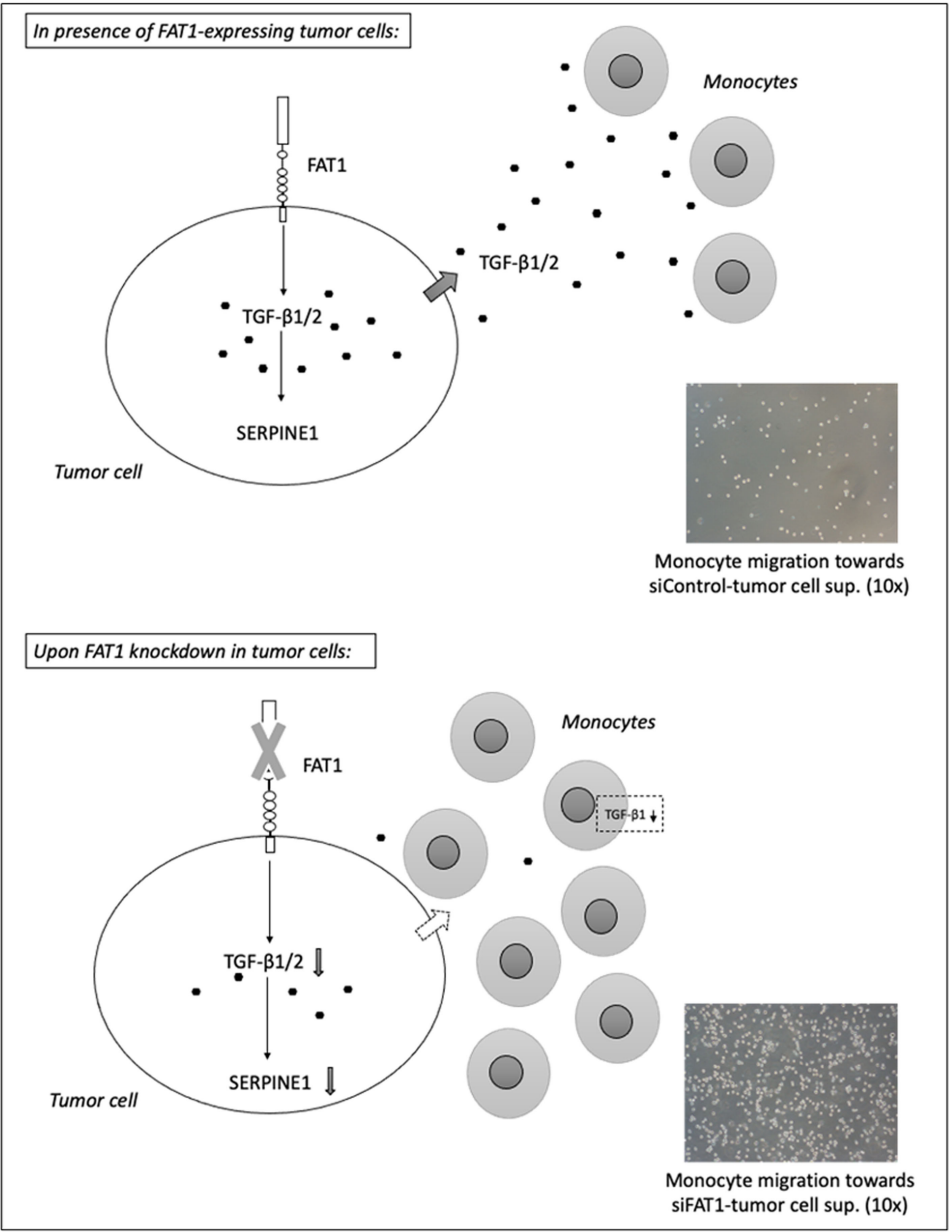
Summary diagram depicts the potential immunosuppressive mechanism mediated by FAT1-expressing tumor cells in a heterogeneous solid tumor microenvironment (*upper panel*). As per TIMER2.0 analysis, FAT1 expression correlates inversely with the infiltration of tumor-inhibiting immune cells in TCGA cancers. The molecular and phenotypic changes in the tumor microenvironment following *FAT1* knockdown in tumor cells have been shown (*lower panel*). As per THP-1 transmigration experiments, decreased TGF-β1 secretion from FAT1-depleted tumor cells may be responsible for increased chemotactic activity of monocytes toward conditioned media of FAT1-depleted tumor cells. When cultured in such conditioned medium, THP-1 monocytes also synthesize reduced TGF-β1, further revealing the contribution of FAT1 in inducing an immunosuppressive milieu.

We found that a significantly higher number of THP-1 monocytes migrated toward conditioned media from siFAT1-transfected U87MG, PC-A, PC-B, and HeLa cells than the number of monocytes that migrated toward conditioned media from the respective siControl cells (**Figures 3A, B**). Thus, increased THP-1 monocyte migration toward conditioned media from siFAT1-transfected tumor cells corresponds to and may be attributed to decreased immunosuppressive cytokine (TGF-β1/TGF-β2) production by FAT1-depleted tumor cells (**Figure 4**).

### Incubation of THP-1 Monocytes in Conditioned Media of FAT1-Depleted Glioma Cells Reduces TGF-β1 Transcript Level in THP-1 Monocytes

THP-1 cells were assessed for changes in TGF-β1 expression upon 24-h incubation in conditioned media (culture supernatants) from siFAT1/siControl-transfected glioma cells (U87MG, PC-A, and PC-B) by q-PCR. TGF-β1 mRNA was found to be decreased in THP-1 cells upon culturing in media from siFAT1-transfected U87MG, PC-A, and PC-B as compared to culture in media from respective siControl cells (**Figure S4**). Thus, apart from FAT1-mediated modulation of immunosuppressive cytokine (TGF-β1) production in the glioma cells, the milieu provided by FAT1-expressing glioma cells may also influence TGF-β1 production in the monocytes present in the microenvironment. This possibly indicates polarization changes in monocytes in the presence of FAT1-expressing glioma cells.

### FAT1 Modulates TGF-β1 Production *via* miR-663a in Cancer Cells

**Figure 5:**
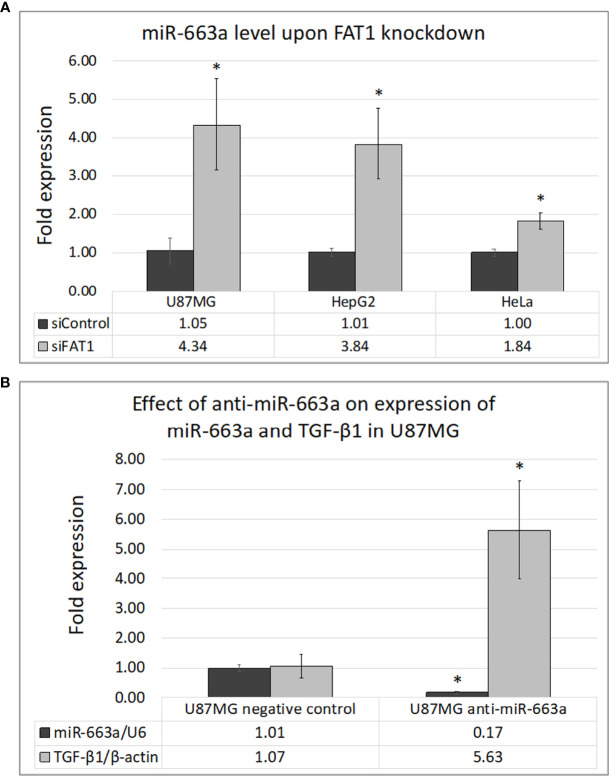
**(A)** Effect of *FAT1* knockdown on the expression of miR-663a in U87MG, HepG2, and HeLa cells as assessed by q-PCR. **(B)** Inhibition of miR-663a in U87MG cells and its effect on the expression of TGF-β1 as assessed by q-PCR. miR-663a expression has been normalized relative to U6 internal control reference, while TGF-β1 expression has been normalized relative to β-actin internal control reference. (*) indicates p-value ≤0.05.

Posttranscriptional regulation of TGF-β1 by miR-663a has recently been shown in hepatocellular carcinoma (40, 41), lung cancer (42), and GBM cells (43). Since the effect of *FAT1* knockdown on TGF-β1 modulation was seen to begin at the mRNA level, we checked whether FAT1 has a role in regulating miR-663a expression in cancer cells. At 72 h of *FAT1* knockdown, U87MG, HepG2, and HeLa cells showed increased levels of miR-663a as compared to siControl cells (**Figure 5A**). In order to confirm the regulation of TGF-β1 transcript by miR-663a, we inhibited miR-663a synthesis in U87MG cells using Anti-miR™ inhibitor of miR-663a. We observed an increase in TGF-β1 mRNA levels in U87MG upon inhibiting miR-663a expression (**Figure 5B**). Thus, our results suggested that FAT1 may possibly promote the translation of TGF-β1 *via* suppression of miR-663a levels, in turn affecting the immunomodulatory properties of cancer cells.

### FAT1 Mediates Immunosuppression in Glioma *via* Myeloid-Derived Suppressor Cells

To study the potential involvement of MDSCs in FAT1-mediated immunosuppression in tumors, we studied the expression of surrogate markers of MDSCs, namely, PD-L1, PD-L2, and IL-10 in gliomas. These genes have been considered as markers to indicate the *in vivo* activity of human MDSCs (27–30) and form a critical component of tumor immunosuppression by inhibition of T-cell activation (44).

**Figure 6:**
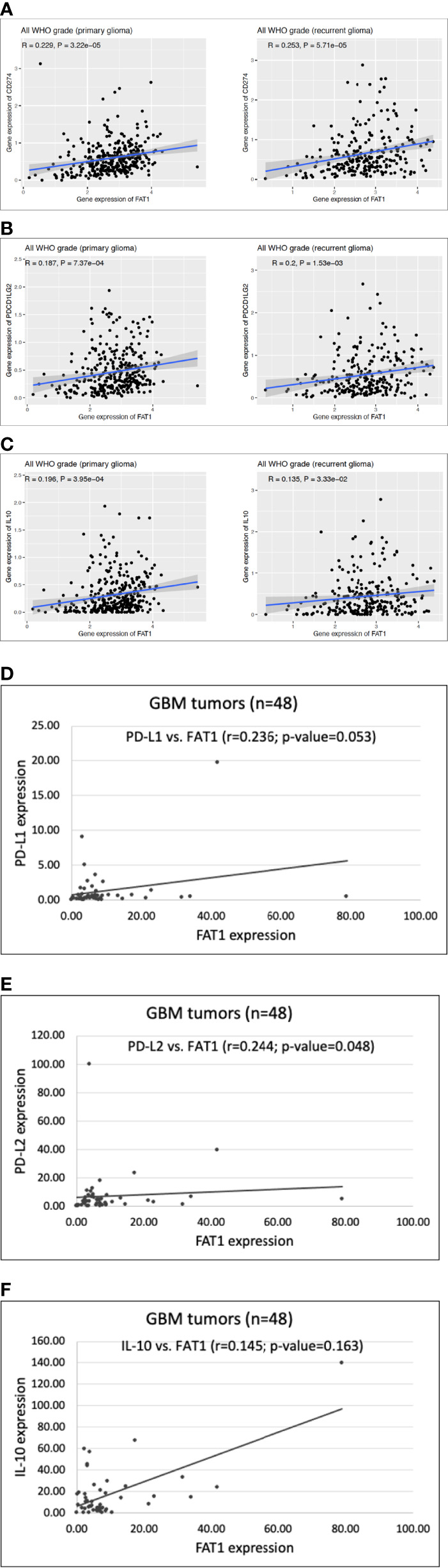
**(A–C)** Scatter plots showing correlation of FAT1 expression with expression of PD-L1 (CD274), PD-L2 (PDCD1LG2) and IL-10 in primary and recurrent glioma cases belonging to CGGA database (dataset: mRNAseq_693). **(D–F)** Scatter plots showing correlation of FAT1 expression with expression of PD-L1, PD-L2 and IL-10 in GBM tumor specimens (n=48) as analysed by q-PCR.

We first did an *in silico* analysis in CGGA database (mRNAseq_693) where we observed significant positive correlations between FAT1 expression and expression of PD-L1, PD-L2, and IL-10 genes in both primary gliomas (PD-L1: r = 0.229, p < 0.01; PD-L2: r = 0.187, p < 0.01; IL-10: r = 0.196, p < 0.01) and recurrent gliomas (PD-L1: r = 0.253, p < 0.01; PD-L2: r = 0.2, p < 0.01; IL-10: r = 0.135, p < 0.05) (**Figures 6A–C**). Following this, we performed q-PCR to analyze these genes using 48 RNA samples that had been extracted from GBM tumor biopsies (**Table S8**). On Spearman’s correlation analysis, significant positive correlations were found between the expression of FAT1 and expression of immunosuppressive markers PD-L1 (r = 0.236, p = 0.053) and PD-L2 (r = 0.244, p = 0.048) in the studied GBM samples (**Figures 6D–F**). Although the correlation between the expression of FAT1 and IL-10 was found to have a positive trend (r = 0.145), unlike the CGGA finding, it was not statistically significant (p = 0.163). This could be due to the small sample size. The expression of IL-10 was found to be undetectable at the mRNA level in the homogeneous *in vitro* cell cultures such as primary glioma cultures and U87MG cell line upon q-PCR analysis (data not shown), thereby indicating the absence of IL-10 production from tumor cells alone. Thus, in most likelihood, the observed IL-10 expression in fresh-frozen GBM tumors indicates the infiltrated immune cells such as MDSCs in the tumor microenvironment.

Hence, upregulated FAT1 in tumor cells positively correlates with increased presence of MDSCs in glioma tumors as indicated by the expression of MDSC surrogate markers. Thus, FAT1 possibly contributes to glioma-associated immunosuppression *via* MDSCs as well, although the exact molecular mechanism involved during the interplay of tumor cells and MDSCs remains to be studied in detail.

## Discussion

*FAT1* gene, located on chromosome 4q35.2, is the human homolog of *Drosophila* gene *fat* and has only been studied in recent years for its role in embryonic development and cancers (1). *FAT1* gene encodes a large transmembrane protein (506 kDa) belonging to an atypical cadherin subfamily. FAT1 protein is composed of extracellular cadherin repeats, EGF-like domains, and laminin G-like domains. Both oncogenic and tumor-suppressive roles of FAT1 are implicated in different cancers and have been attributed to tissue specificity or context specificity (1). While cancers of skin, lung, head and neck, and mouth display a tumor-suppressive function (4–6) of FAT1, other cancers such as hepatocellular carcinoma, cervical cancer, pancreatic cancer, and gliomas (3, 7–13) are known to show an oncogenic role of FAT1. These tissue type-dependent differences warrant a deeper understanding of the molecular milieu that could determine the context-dependent changes in gene function. Our lab has previously reported the oncogenic role of FAT1 in glioma *via* regulation of migration, invasion, and stemness (11–13). In the present study, we have found an oncogenic role of FAT1 *via* modulation of immunosuppression using primary cultures derived from surgical gliomas and the U87MG cell line. The other cancer cell lines used in this study, namely, HepG2, Panc-1, and HeLa, are representative of liver, pancreatic, and cervical cancers, respectively, which have also been shown by other groups to display an oncogenic role of FAT1 (2, 8–10).

TGF-β cytokines are key players responsible for immunosuppression in solid cancers such as high-grade glioma (25), hepatocellular carcinoma (45), cervical cancer (46), pancreatic cancer (47), and colorectal cancer (48). In the GBM microenvironment especially, TGF-β is known to be secreted from glioma cells *via* autocrine mechanism and from infiltrating microglial cells (49). Autocrine secretion of TGF-β has been noted in both glioma cell lines and in cells procured from surgically resected gliomas (49). As a relatively new gene implicated in tumorigenesis, FAT1 has not yet been studied in relation to the expression and secretion of immunosuppressive mediators.

Although, in humans, three TGF-β isoforms are known to be expressed—TGF-β1, TGF-β2, and TGF-β3—TGF-β1 is the most abundant isoform (50). TGF-β1 mRNA has been found to be upregulated in tumor tissues of several cancer types that has also shown a correlation with advanced tumor stage and poor prognosis (38, 51). Functional studies have further elaborated the role of increased TGF-β1 expression in promoting malignant phenotypes such as EMT, stemness, angiogenesis, invasion, and metastasis (51). On the other hand, TGF-β2 was originally described in human GBM cells, while it is physiologically known to be expressed in neurons, astroglia, and cells of embryonic central nervous system (25, 50). In gliomas, the expression of both TGF-β1 and TGF-β2 has been observed to be higher in tumor samples as compared to non-tumoral brain samples (52).

Aberrant upregulation of TGF-β signaling has been previously noted in GBM, while Serpine1 (PAI-1) has been described as a classical marker of TGF-β pathway activation in GBM (36). In fact, glioma patients displaying a high expression of Serpine1 in biopsies have been known to show shorter overall survival (53). In other tumors as well, such as hepatocellular carcinoma, pancreatic ductal adenocarcinoma, and cervical cancer, high Serpine1 expression has been associated with a poor clinical outcome (54–56).

Canonically, intracellular TGF-β signaling is triggered by the binding of TGF-β family ligands to type I and type II receptors on the cellular surface (57). Type III receptor is a co-receptor that facilitates interaction of the ligand with type I and type II receptors. Ligand–receptor binding induces phosphorylation of mothers against decapentaplegic (SMAD)2/3 proteins, which form a complex with SMAD4 and translocate into the nucleus to regulate target gene expression. Serpine1 is a typical target gene induced downstream of SMAD3 phosphorylation in the canonical pathway (57). Thus, SMAD2/3 phosphorylation and Serpine1 upregulation in cells may serve as effective readouts of elevated TGF-β levels. For example, in fibrotic diseases, it has been seen that elevated levels of TGF-β correspond to fibroblasts displaying activation of TGF-β signaling with nuclear accumulation of active SMAD3 and increased transcription of Serpine1 (58). However, it may be noted that Serpine1 gene is also known to be regulated by nuclear factor kappa B (NF-κB) (RelA), p53, and TEL2 transcription factors in other cancer cells such as osteosarcoma and nasopharyngeal carcinoma (59, 60).

Our preliminary TIMER2.0 analysis in TCGA data revealed an inverse correlation between FAT1 expression and infiltration levels of tumor-inhibiting immune cells in glioma, cervical cancer, liver cancer, and pancreatic cancer. The presence of tumor-infiltrating lymphocytes (TILs) is known to be predictive of improved clinical outcome, as tumors are immunogenic and elicit immune surveillance against cell transformation (61–63).

We next analyzed transcript levels of immunosuppressive mediators TGF-β1 and TGF-β2 along with FAT1 in fresh-frozen GBM tissues using q-PCR. We found a positive correlation of FAT1 expression with that of TGF-β1 and TGF-β2, as confirmed by Spearman’s test and heat map analysis. For *in silico* validation, we correlated the expression of TGF-β1 and TGF-β2 genes with FAT1 expression in cases belonging to different cancers using TCGA and GLASS databases. We noted a positive association between FAT1 expression and expression of TGF-β1/TGF-β2 in glioma and cancers of the liver, cervix, pancreas, and colon in these databases. Following the patient sample study, we transiently silenced *FAT1* gene in various *in vitro* cancer cell lines and primary cultures of surgical gliomas. This resulted in a decrease in the expression of TGF-β1, TGF-β2, and downstream effectors of activated TGF-β signaling (Serpine1 and SMAD2/3) in most of the studied cultures. We also noted decreased secretion of TGF-β1 in response to *FAT1* knockdown in case of siFAT1-treated cultures of glioma cells, HeLa cells, and HepG2 cells using an ELISA array.

At extremely low concentrations, TGF-β is known to direct monocyte migration *in vitro* that can be assayed using Transwell inserts (64). Consequently, we found that using an *in vitro* THP-1 monocyte chemotaxis model (34) that decreased TGF-β1/TGF-β2 production by FAT1-depleted tumor cells led to increased migration of THP-1 monocytes toward supernatants of siFAT1-treated cells. It is well-known that microglia are a predominant part of resident immune cells in the brain, which form infiltrating tumor-associated macrophages (TAMs) in the GBM microenvironment (65). Our transmigration results provide a functional relevance of the reduction found in immunosuppressive cytokines in response to *FAT1* knockdown in tumor cells.

IL-6 is known to promote glioma cell proliferation and invasion (66). In fact, potential IL-6 inhibitors have been evaluated extensively for therapeutic efficacy against various cancers (67). Our ELISA results showed decreased secretion of IL-6 from glioma cells in response to *FAT1* knockdown. This leads us to deduce that FAT1 probably employs both cell growth-promoting features of some cytokines (such as IL-6) and immunity-evading properties of others (such as TGF-β) to the advantage of the tumor.

We next observed that brief incubation of THP-1 monocytes (24 h) in conditioned media from siFAT1-treated glioma cells resulted in decreased TGF-β1 expression in THP-1 cells. This indicates some secretory product in high FAT1-expressing glioma cell supernatant that affects the polarization of THP-1 cells (19, 68). The nature of the product, however, needs to be elucidated further. Our result hints at a polarization change in THP-1 monocytes in the presence of siFAT1–glioma cell supernatant, pointing to skewing of M2 phenotype of monocytes to M1. Hence, it is possible that in a heterotypic tumor microenvironment, especially in glioma, FAT1 influences TGF-β expression in more than one cell type, thereby contributing to an immunosuppressive milieu.

To further investigate the possible molecular mechanism connecting FAT1 with regulation of TGF-β, we checked siFAT1-treated cells for changes in the level of miR-663a, which has previously been reported to posttranscriptionally regulate TGF-β1 in glioma and hepatocellular carcinoma cells (40, 41, 43). We found enhanced miR-663a levels in U87MG, HepG2, and HeLa cells upon *FAT1* knockdown as compared to siControl cells, indicating a role of FAT1 in modulating miR-663a that might indirectly regulate TGF-β1 expression in tumor cells.

In addition, we also explored the potential involvement of MDSCs in FAT1-mediated immunosuppression in gliomas. We obtained a positive correlation of FAT1 expression with the expression of IL-10, PD-L1, and PD-L2 genes, the known surrogate markers of MDSCs, in primary and recurrent CGGA glioma cases. In fresh-frozen GBM samples, q-PCR of these genes yielded a positive correlation of FAT1 expression with PD-L1 and PD-L2 immune checkpoints. Hence, as suggested by the markers of MDSCs, increased expression of FAT1 in tumor cells may also correspond with increased MDSC infiltration, thereby contributing to another mode of immunosuppression in gliomas. Similar observations were also made in TIMER2.0 analysis where we had found a positive correlation between FAT1 expression and infiltration levels of tumor-promoting MDSCs in TCGA cases of cervical cancer, GBM, and pancreatic cancer.

The functional association between FAT1 and TGF-β1/TGF-β2 cytokines and the association of FAT1 with IL-10 cytokine and PD-L1/L2 immune checkpoints have not been reported in cancers so far. Our study unravels a novel function of FAT1 in the maintenance of immunosuppressive tumor microenvironment. FAT1 is overexpressed in a subset of GBM tumors. We have earlier reported that an upregulated FAT1 promotes protumorigenic inflammation in glioma *via* the AP1 pathway, leading to increased cytokines such as IL-6 and IL-1β and also increased COX-2 (11). This also results in increased hypoxia inducible factor (HIF)-1α in a hypoxic microenvironment (12), as also increased stemness and epithelial to mesenchymal transition (EMT) (13). Hence, high levels of this atypical cadherin would affect both the tumor and the microenvironment, giving rise to conditions conducive to an adverse tumor phenotype both intrinsically and by immune suppression.

## Data Availability Statement

The original contributions presented in the study are included in the article/**Supplementary Material**. Further inquiries can be directed to the corresponding authors.

## Ethics Statement

The studies involving human participants were reviewed and approved by the Institute Ethics Committee, AIIMS, New Delhi (Ref no. IECPG-3/28.10.2015, RT-16/30.12.2015; IECPG/184/2/2019). Written informed consent to participate in this study was provided by the participants’ legal guardian/next of kin.

## Author Contributions

KI, CSr, NM, MA, YG, and SG performed the experiments, data acquisition, and result analysis. CSa, VS, and SM performed the histological diagnosis and classification of patient samples. DG and AS performed surgery and provided clinical samples. KI, KC, SS, and PC contributed to study design, article preparation and editing, data evaluation, funding, and resources. All the authors have read and approved the final article.

## Funding

This work has been financially supported by the SERB Start-Up Grant from the Science and Engineering Research Board (DST-SERB), India [No. YSS/2015/001613], and DHR Young Scientist Grant from the Department of Health Research (DHR-ICMR), India [No. R.12014/11/2019-HR] to KI; DBT grant [BT/PR13357/MED/30/1532/2015] and DST grant [SR/FT/LS-126/2010] to KC; JC Bose fellowship of DST, India, to SS; AIIMS fellowship to CSr; ICMR fellowship to NM and YG; DBT fellowship to MA; and CSIR fellowship to SG.

## Conflict of Interest

The authors declare that the research was conducted in the absence of any commercial or financial relationships that could be construed as a potential conflict of interest.

## Publisher’s Note

All claims expressed in this article are solely those of the authors and do not necessarily represent those of their affiliated organizations, or those of the publisher, the editors and the reviewers. Any product that may be evaluated in this article, or claim that may be made by its manufacturer, is not guaranteed or endorsed by the publisher.

## Abbreviations

GBM, glioblastoma; TGF-β1, transforming growth factor-β1; PD-L1, programmed death ligand-1; TCGA, The Cancer Genome Atlas; GLASS, Glioma Longitudinal Analysis Consortium; CGGA, Chinese Glioma Genome Atlas; TIMER2.0, Tumor IMmune Estimation Resource 2.0.
