# Supplementary material for: Upregulation of Atypical Cadherin FAT1 Promotes an Immunosuppressive Tumor Microenvironment via TGF-β
Source: Front Immunol. 2022 May 26;13:813888. doi: 10.3389/fimmu.2022.813888 (PMC9205206; doi:10.3389/fimmu.2022.813888)
Supplement: Supplementary file 1 [file DataSheet_1.pdf]

Figure S1(a)

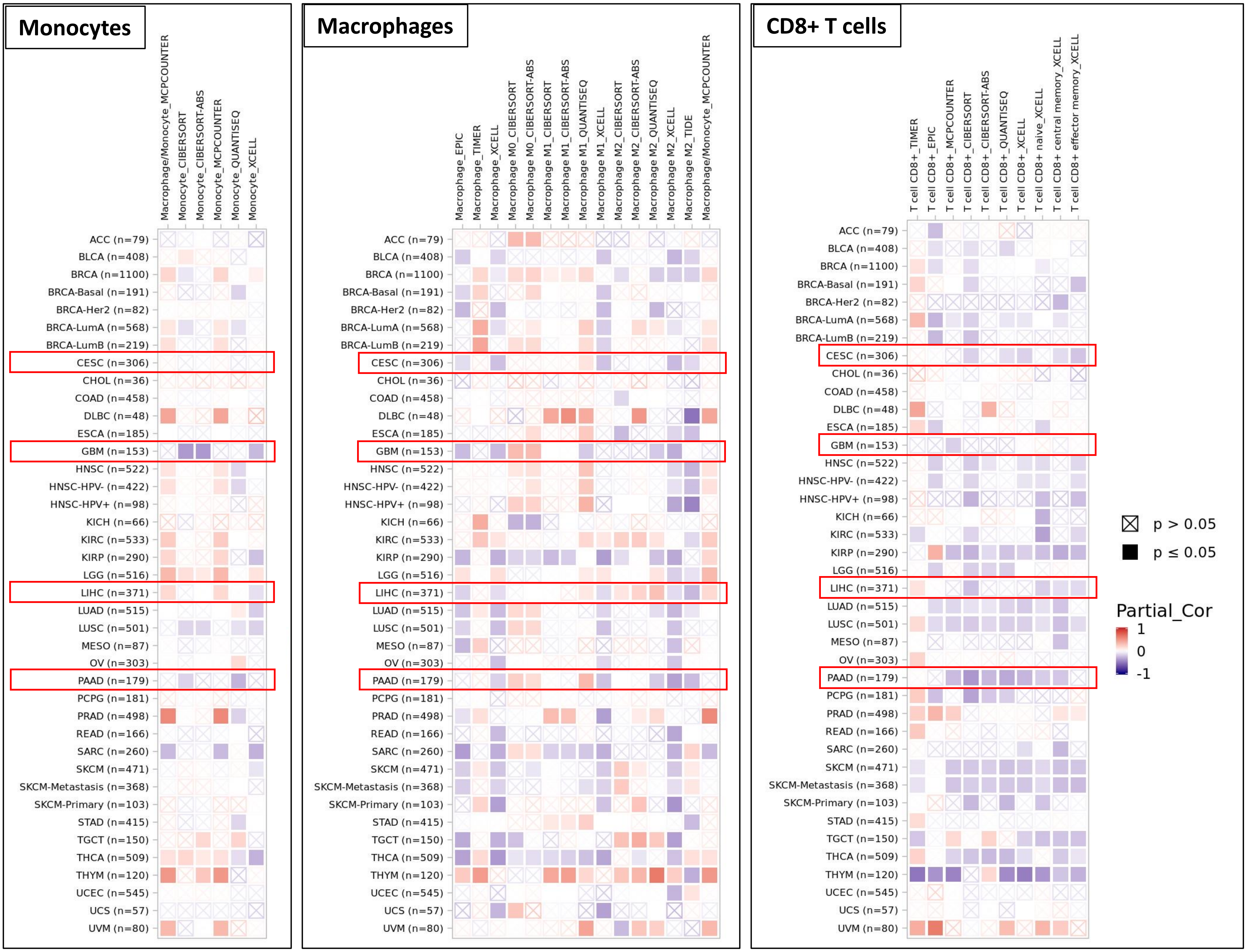

Figure S1. (a-c) Heat maps generated by TIMER2.0 depicting the correlation between FAT1 gene expression and infiltration levels of different types of immune cells in cancer cases from TCGA database. CESC (cervical and endocervical cancer), GBM (glioblastoma), LIHC (liver hepatocellular carcinoma) and PAAD (pancreatic adenocarcinoma) have been highlighted in the heat maps using red rectangles. Different algorithms such as xCell, MCP-counter, EPIC, quanTIseq, etc. are applied by TIMER2.0 web server to the expression profiles of TCGA tumors for immune infiltration estimation.

Figure S1(b)

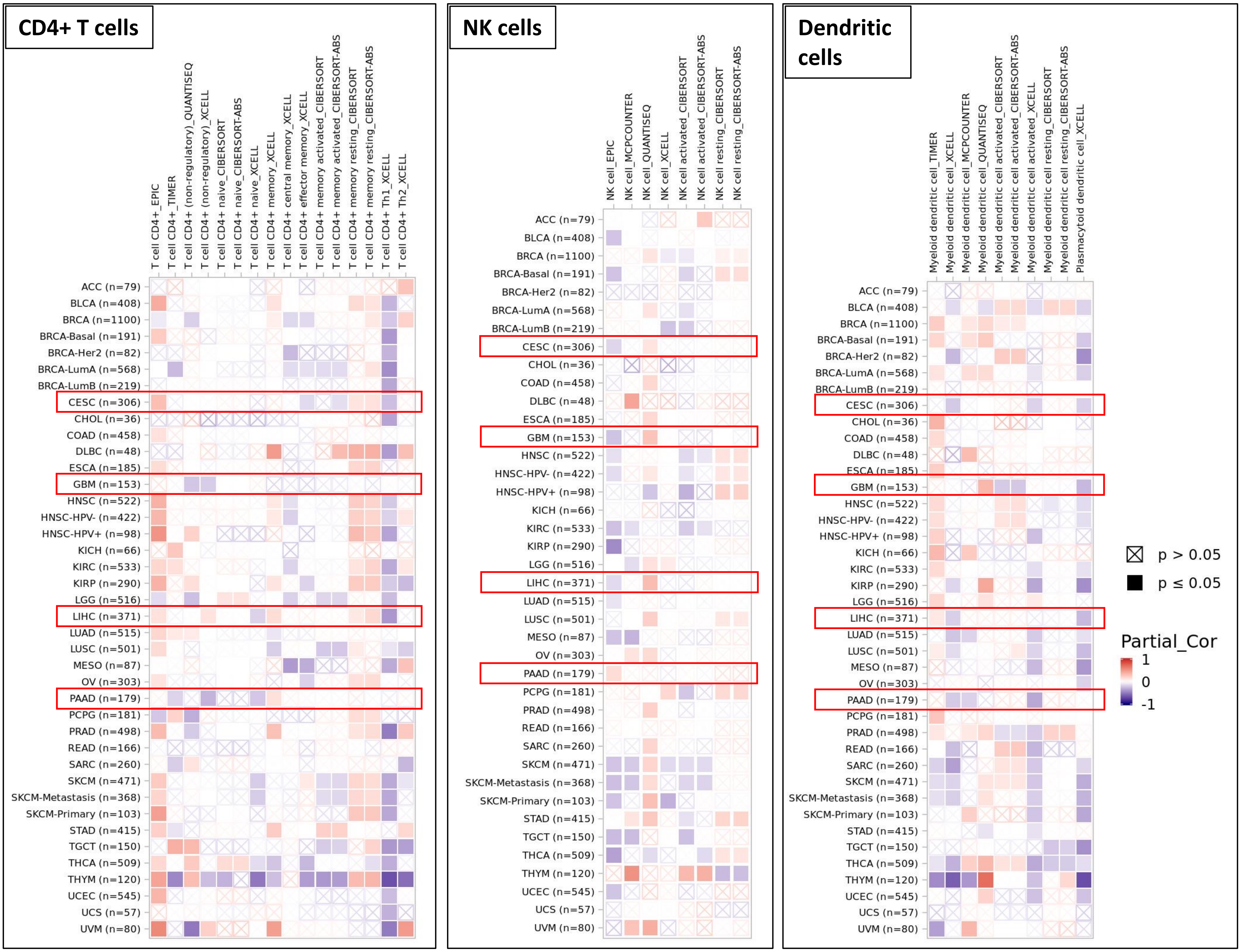

CD4+ T cells
NK cells
Dendritic cells
p > 0.05
p ≤ 0.05
Partial_Cor
1
0
-1

Figure S1(c)

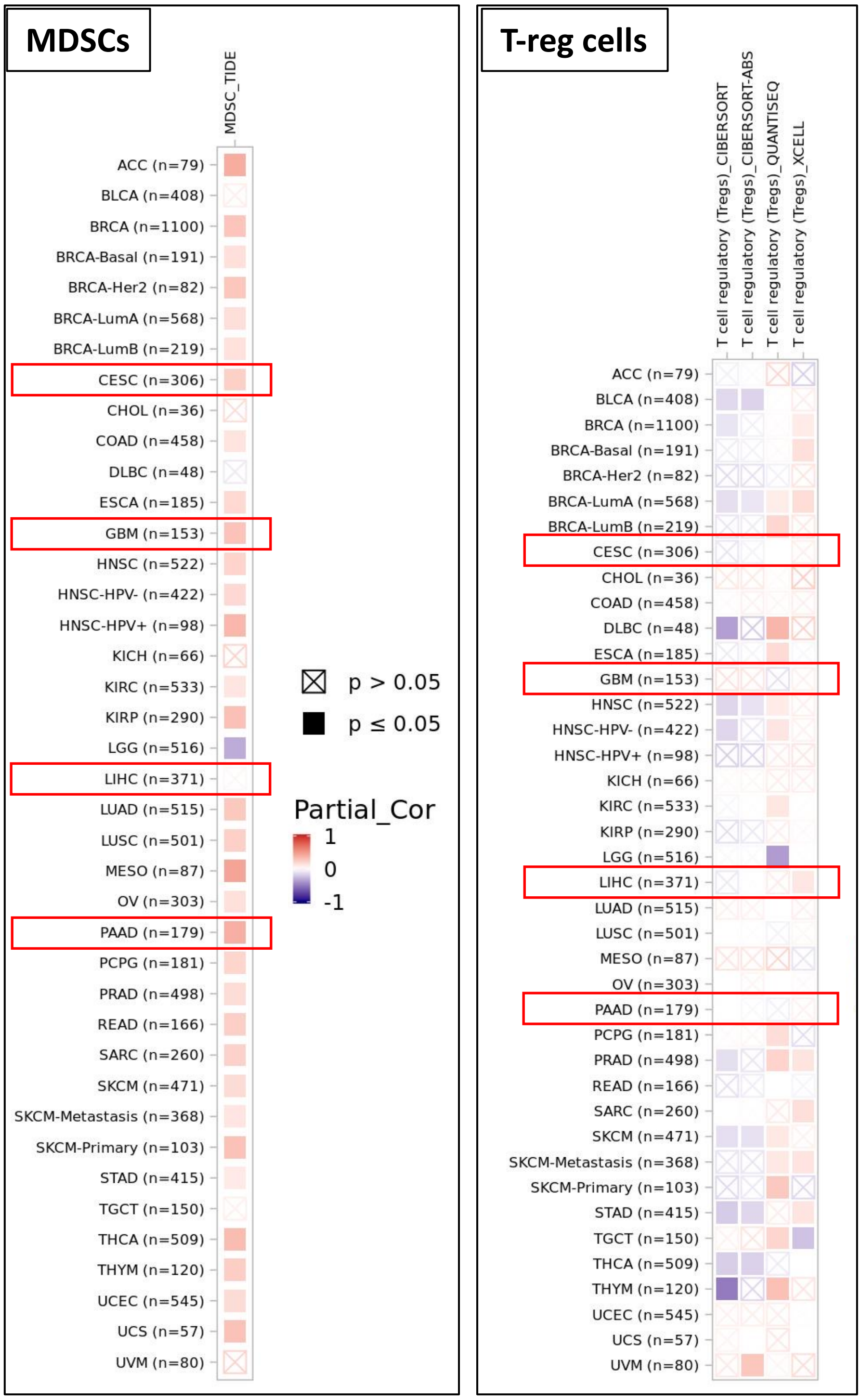
MDSCs
T-reg cells
MDSC_TIDE
T cell regulatory (Tregs)_CIBERSORT
T cell regulatory (Tregs)_CIBERSORT-ABS
T cell regulatory (Tregs)_QUANTISEQ
T cell regulatory (Tregs)_XCELL
ACC (n=79)
BLCA (n=408)
BRCA (n=1100)
BRCA-Basal (n=191)
BRCA-Her2 (n=82)
BRCA-LumA (n=568)
BRCA-LumB (n=219)
CESC (n=306)
CHOL (n=36)
COAD (n=458)
DLBC (n=48)
ESCA (n=185)
GBM (n=153)
HNSC (n=522)
HNSC-HPV- (n=422)
HNSC-HPV+ (n=98)
KICH (n=66)
KIRC (n=533)
KIRP (n=290)
LGG (n=516)
LIHC (n=371)
LUAD (n=515)
LUSC (n=501)
MESO (n=87)
OV (n=303)
PAAD (n=179)
PCPG (n=181)
PRAD (n=498)
READ (n=166)
SARC (n=260)
SKCM (n=471)
SKCM-Metastasis (n=368)
SKCM-Primary (n=103)
STAD (n=415)
TGCT (n=150)
THCA (n=509)
THYM (n=120)
UCEC (n=545)
UCS (n=57)
UVM (n=80)
p > 0.05
p ≤ 0.05
Partial_Cor
1
0
-1

Figure S2

(a)

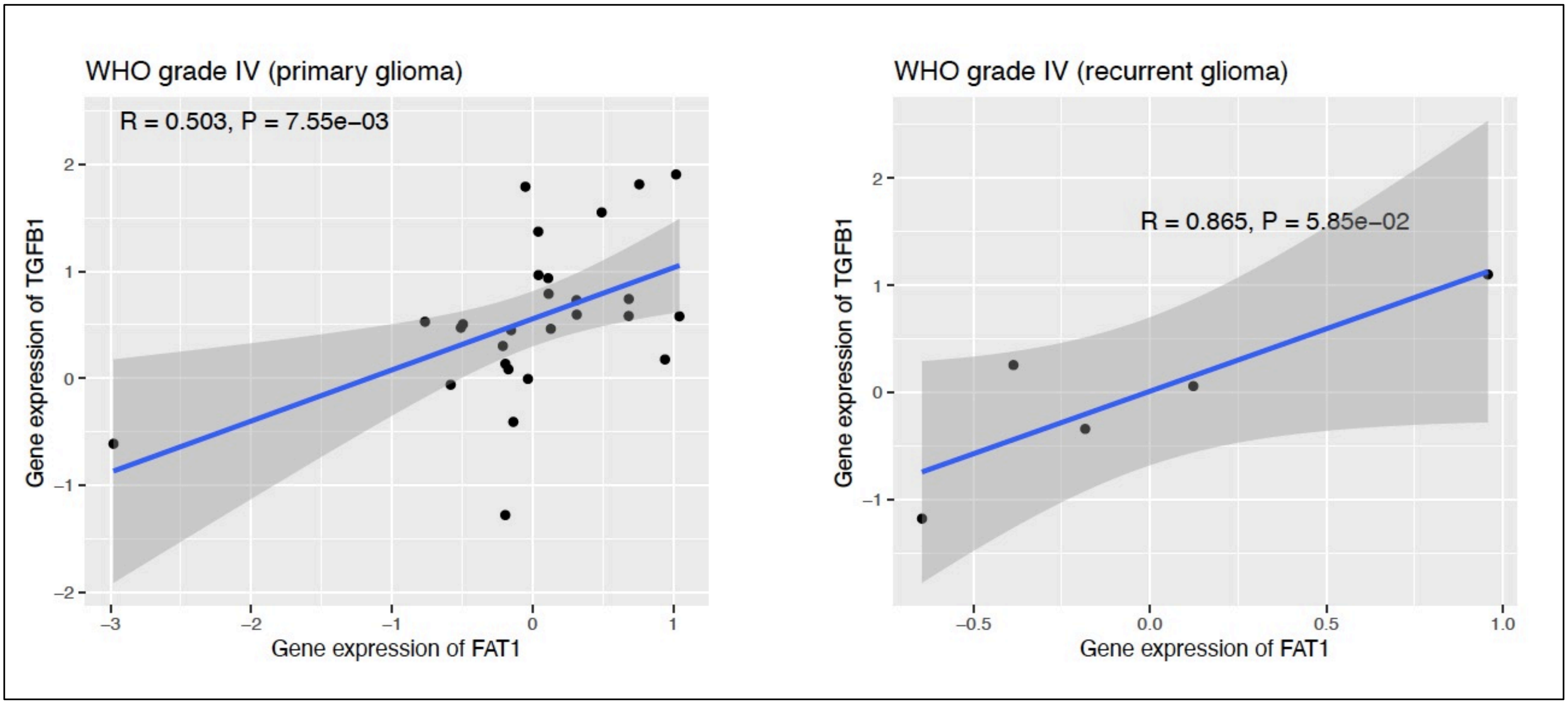

(b)

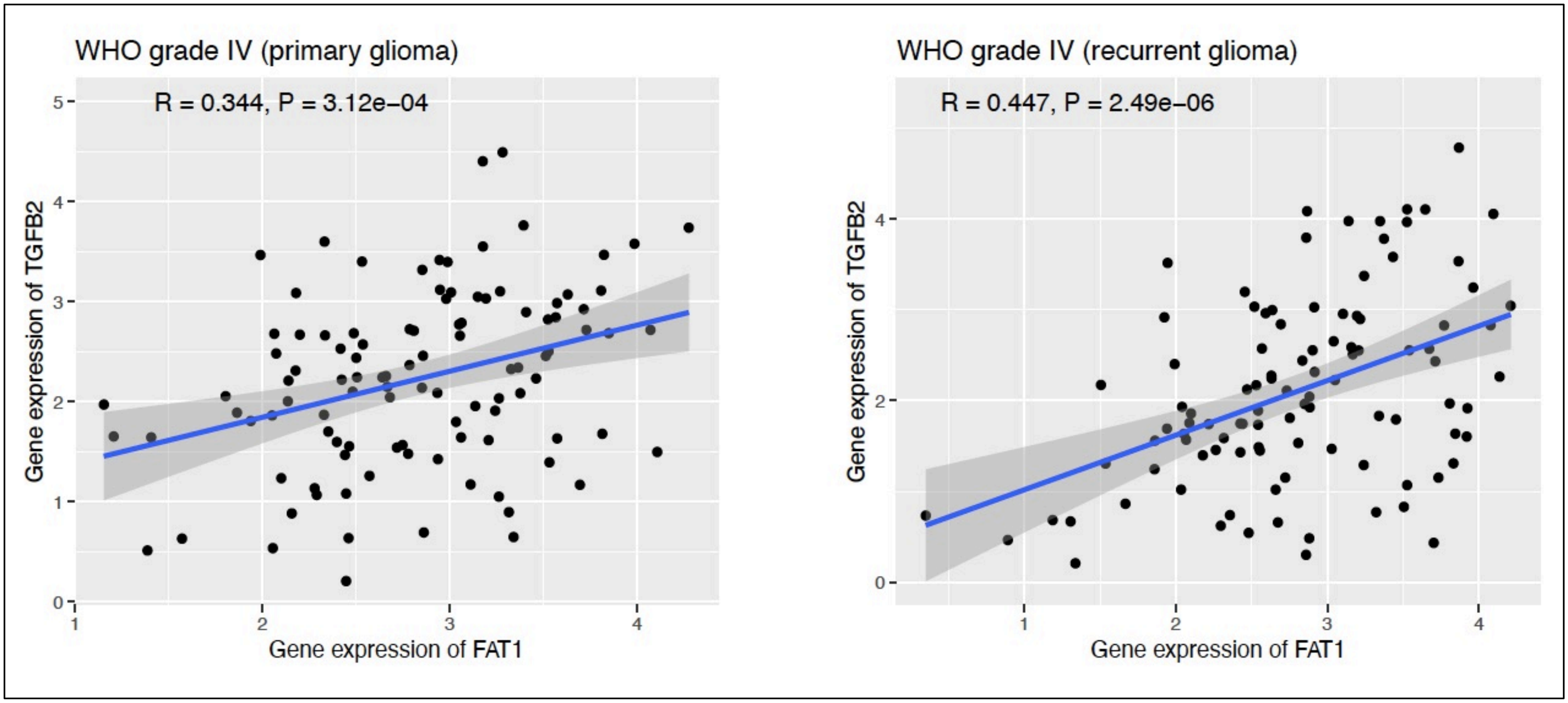

Figure S2. (a-b) Scatter plots showing correlation of FAT1 expression with expression of TGF-β1 (mRNA_array_301 dataset) and TGF-β2 (mRNAseq_693 dataset) in primary and recurrent glioma cases belonging to CGGA database.

Figure S3

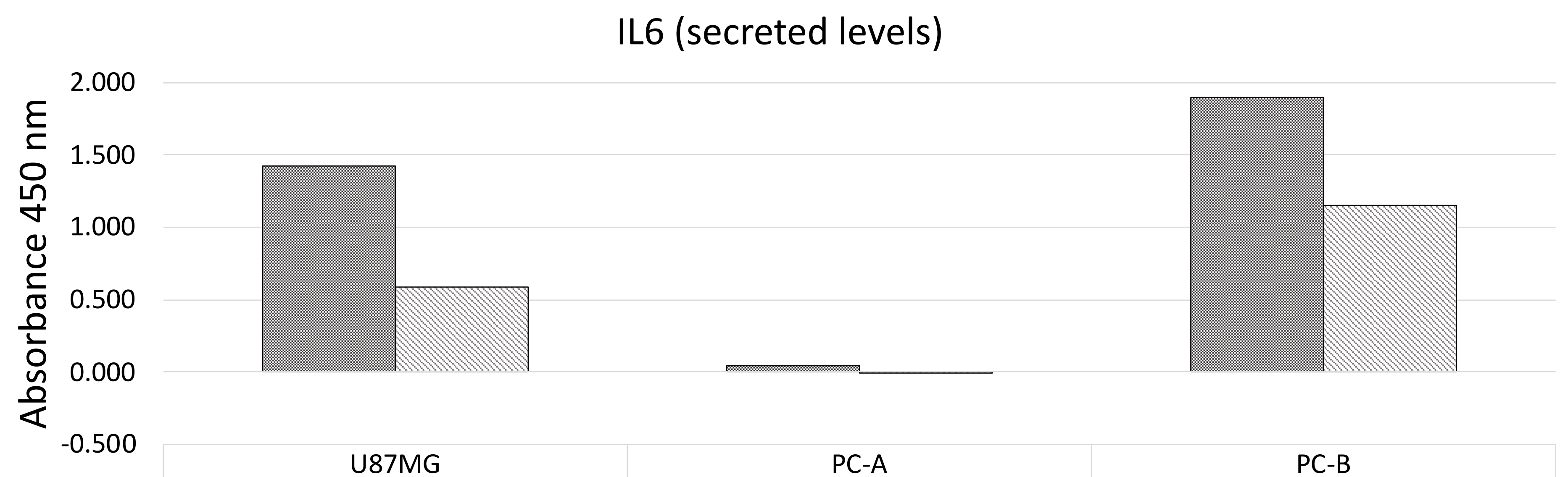

	U87MG	PC-A	PC-B
siControl	1.423	0.037	1.900
siFAT1	0.584	-0.004	1.152

Figure S3. Graph showing optical densities of IL-6 cytokine secreted by siControl cells versus siFAT1 cells in U87MG, PC-A and PC-B culture supernatants depicting modulation of cytokine release by tumor cells upon *FAT1* knockdown.

Figure S4

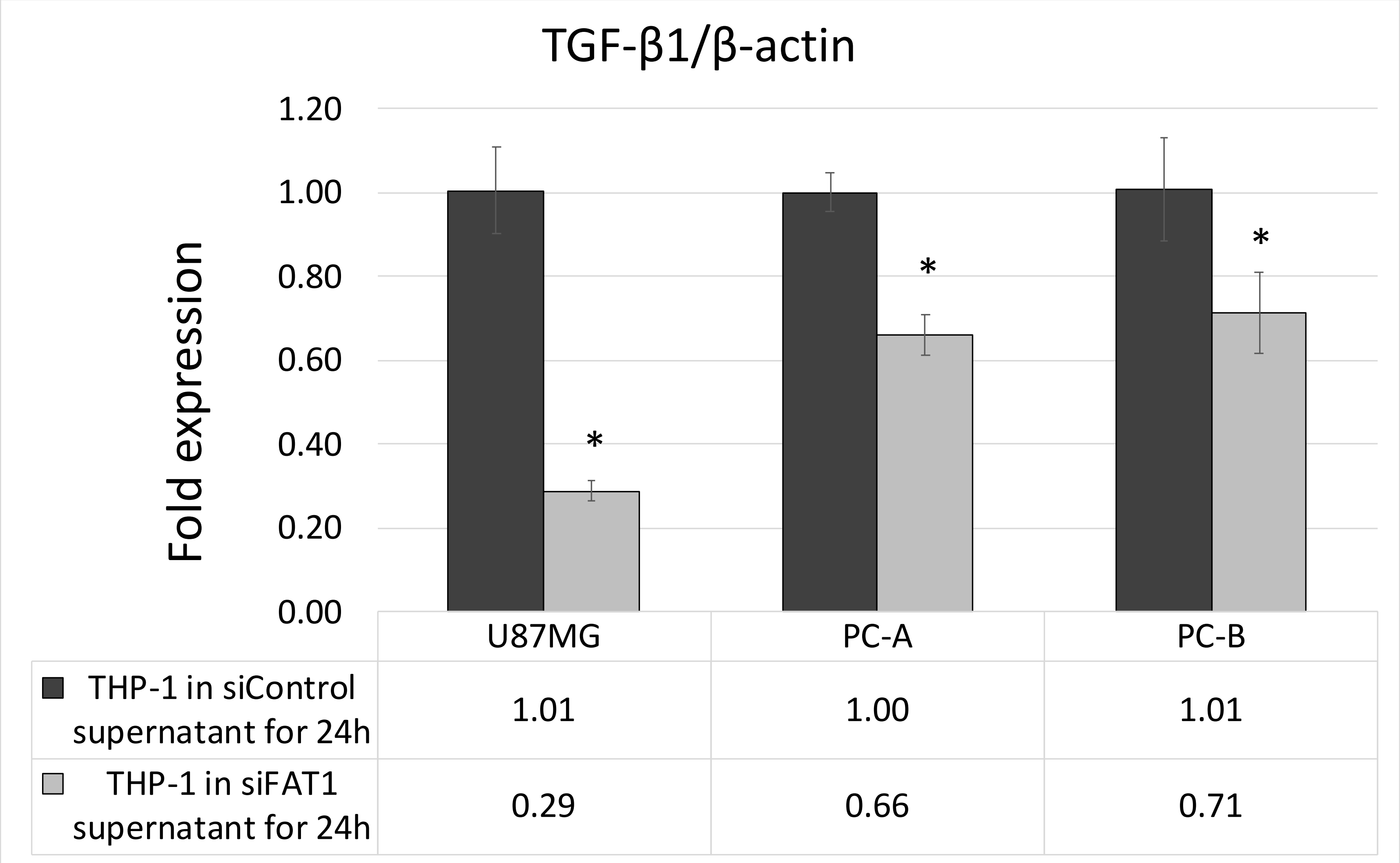

	U87MG	PC-A	PC-B
THP-1 in siControl supernatant for 24h	1.01	1.00	1.01
THP-1 in siFAT1 supernatant for 24h	0.29	0.66	0.71

Figure S4. Graph showing mRNA expression of TGF-β1 in THP-1 cells in response to 24-hour incubation in conditioned media from siFAT1/siControl-transfected U87MG, PC-A and PC-B glioma cells as analysed by q-PCR. (*) indicates p-value ≤0.05.
