# Supplementary material for: Upregulation of Atypical Cadherin FAT1 Promotes an Immunosuppressive Tumor Microenvironment via TGF-β
Source: Front Immunol. 2022 May 26;13:813888. doi: 10.3389/fimmu.2022.813888 (PMC9205206; doi:10.3389/fimmu.2022.813888)
Supplement: Supplementary file 2 [file DataSheet_2.pdf]

Supplementary tables

Table S1: Details of primary cultures generated from fresh surgical glioma tissues

**Primary cultures (PC) successfully generated**	**FAT1 expression w.r.t. U87MG (±SD)**	**Time from P0 to 1st subculture**	**Passage at siFAT1 experiment**
PC-A: Oligodendroglioma (Grade II)	PC-A/U87MG = 4.6-fold (±0.73)	48 days	P5 (day 82)
PC-B: GBM (Grade IV)	PC-B/U87MG = 6.3-fold (±1.05)	26 days	P3 (day 58)

Table S2: List of primers used for q-PCR analysis

Gene	Primer	Sequence (5'-3')
FAT1	Forward	5' AAAATAGGTGAAGAGACAGGTGT 3'
	Reverse	5' TCTGTGGTGCATTGTCATTGA 3'
TGF-β1	Forward	5' CCCTGGACACCAACTATTGC 3'
	Reverse	5' GTCCAGGCTCCAAATGTAGG 3'
TGF-β2	Forward	5' ATCCCGCCCACTTTCTACAG 3'
	Reverse	5' CGCTGGGTTGGAGATGTTAA 3'
SERPINE1	Forward	5' TATACTGAGTTCACCACGCC 3'
	Reverse	5' GTCATGTTGCCTTTCCAGTG 3'
IL-10	Forward	5' GCCAAGCCTTGTCTGAGATG 3'
	Reverse	5' AAGAAATCGATGACAGCGCC 3'
PD-L1	Forward	5' TATGGTGGTGCCGACTACAA 3'
	Reverse	5' TGCTTGTCCAGATGACTTCG 3'
PD-L2	Forward	5' GGGACGAAGGACAGTACCAA 3'
	Reverse	5' TTTGGCCAGGATACTTCTGC 3'
18S rRNA	Forward	5' GTAACCCGTTGAACCCCATT 3'
	Reverse	5' CCATCCAATCGGTAGTAGCG 3'
β-actin	Forward	5' TCATGAAGTGTGACGTGGACATCCGC 3'
	Reverse	5' CCTAGAAGCATTTGCGGTGGACGATG 3'

Table S3: Fold expression values of FAT1 and TGF-β signaling genes in 49 GBM samples calculated with respect to normal brain levels and normalized by 18S rRNA internal control.

GBM ID	FAT1	TGF-β1	TGF-β2	SERPINE1
GBM 1	0.64	0.09	0.70	0.65
GBM 2	2.80	0.34	3.04	22.26
GBM 3	4.52	1.24	0.20	117.50
GBM 4	10.63	0.86	3.07	7.52
GBM 5	2.61	0.92	0.94	1.50
GBM 6	0.59	0.23	0.32	4.33
GBM 7	5.35	2.98	0.44	16.12
GBM 8	0.03	0.01	0.03	0.38
GBM 9	7.01	0.42	2.20	2.26
GBM 10	2.07	0.30	2.10	3.69
GBM 11	7.62	1.47	1.60	3.68
GBM 12	2.32	1.08	2.02	11.67
GBM 13	0.34	0.11	0.31	2.84
GBM 14	3.18	1.83	5.81	28.16
GBM 15	3.18	1.88	3.26	182.36
GBM 16	3.74	1.13	18.03	204.25
GBM 17	6.99	1.63	11.52	27.44
GBM 18	17.35	1.00	2.98	201.05
GBM 19	34.13	1.59	8.21	42.82
GBM 20	8.78	0.08	0.42	6.24
GBM 21	41.88	0.24	21.83	531.08
GBM 22	8.84	1.88	5.06	15.06
GBM 23	5.05	1.45	2.47	18.92
GBM 24	7.14	4.50	8.41	67.66
GBM 25	4.71	0.94	3.04	4.13
GBM 26	3.12	1.01	0.63	0.00
GBM 27	7.83	1.42	0.32	0.82
GBM 28	3.91	1.75	30.75	67.45
GBM 29	7.07	1.59	4.96	15.12
GBM 30	2.78	2.47	21.27	38.70
GBM 31	1.56	0.66	0.90	14.29
GBM 32	8.59	11.62	27.11	13.73
GBM 33	9.22	16.04	7.02	84.94
GBM 34	6.83	1.71	8.26	30.09
GBM 35	3.83	2.08	8.04	162.46
GBM 36	78.96	8.79	109.82	418.94
GBM 37	16.18	7.66	19.29	214.02
GBM 38	14.64	19.78	6.39	57.53
GBM 39	4.40	1.63	11.63	141.20
GBM 40	6.12	0.38	0.32	0.49
GBM 41	6.00	0.09	0.28	63.13
GBM 42	23.00	5.13	10.78	124.92
GBM 43	21.43	13.64	194.53	128.01
GBM 44	6.39	0.00	6.21	54.90
GBM 45	6.23	0.43	0.65	12.50
GBM 46	2.54	0.18	2.21	14.68
GBM 47	13.31	5.06	8.41	409.16
GBM 48	3.59	1.16	0.47	6.40
GBM 49	31.58	9.68	13.64	259.09

Table S4: Spearman's correlation between FAT1 and anti-inflammatory signaling genes in 49 GBM tumors

**Correlations**						
			**FAT1**	**TGF-β1**	**TGF-β2**	**SERPINE1**
Spearman's rho	**FAT1**	Correlation Coefficient	1.000	.517**	.523**	.512**
		Sig. (1-tailed)		.000	.000	.000
	**TGF-β1**	Correlation Coefficient	.517**	1.000	.659**	.555**
		Sig. (1-tailed)	.000		.000	.000
	**TGF-β2**	Correlation Coefficient	.523**	.659**	1.000	.703**
		Sig. (1-tailed)	.000	.000		.000
	**SERPINE1**	Correlation Coefficient	.512**	.555**	.703**	1.000
		Sig. (1-tailed)	.000	.000	.000	
**Correlation is significant at the 0.01 level (1-tailed).						
*Correlation is significant at the 0.05 level (1-tailed).						

Table S5: Fold expression values of TGF-β signaling genes arranged in decreasing order of FAT1 expression for tertile analysis in 49 GBM samples

GBM ID	FAT1	TGF-β1	TGF-β2	SERPINE1
GBM 36	78.96	8.79	109.82	418.94
GBM 21	41.88	0.24	21.83	531.08
GBM 19	34.13	1.59	8.21	42.82
GBM 49	31.58	9.68	13.64	259.09
GBM 42	23.00	5.13	10.78	124.92
GBM 43	21.43	13.64	194.53	128.01
GBM 18	17.35	1.00	2.98	201.05
GBM 37	16.18	7.66	19.29	214.02
GBM 38	14.64	19.78	6.39	57.53
GBM 47	13.31	5.06	8.41	409.16
GBM 4	10.63	0.86	3.07	7.52
GBM 33	9.22	16.04	7.02	84.94
GBM 22	8.84	1.88	5.06	15.06
GBM 20	8.78	0.08	0.42	6.24
GBM 32	8.59	11.62	27.11	13.73
GBM 27	7.83	1.42	0.32	0.82
GBM 11	7.62	1.47	1.60	3.68
GBM 24	7.14	4.50	8.41	67.66
GBM 29	7.07	1.59	4.96	15.12
GBM 9	7.01	0.42	2.20	2.26
GBM 17	6.99	1.63	11.52	27.44
GBM 34	6.83	1.71	8.26	30.09
GBM 44	6.39	0.00	6.21	54.90
GBM 45	6.23	0.43	0.65	12.50
GBM 40	6.12	0.38	0.32	0.49
GBM 41	6.00	0.09	0.28	63.13
GBM 7	5.35	2.98	0.44	16.12
GBM 23	5.05	1.45	2.47	18.92
GBM 25	4.71	0.94	3.04	4.13
GBM 3	4.52	1.24	0.20	117.50
GBM 39	4.40	1.63	11.63	141.20
GBM 28	3.91	1.75	30.75	67.45
GBM 35	3.83	2.08	8.04	162.46
GBM 16	3.74	1.13	18.03	204.25
GBM 48	3.59	1.16	0.47	6.40
GBM 14	3.18	1.83	5.81	28.16
GBM 15	3.18	1.88	3.26	182.36
GBM 26	3.12	1.01	0.63	0.00
GBM 2	2.80	0.34	3.04	22.26
GBM 30	2.78	2.47	21.27	38.70
GBM 5	2.61	0.92	0.94	1.50
GBM 46	2.54	0.18	2.21	14.68
GBM 12	2.32	1.08	2.02	11.67
GBM 10	2.07	0.30	2.10	3.69
GBM 31	1.56	0.66	0.90	14.29
GBM 1	0.64	0.09	0.70	0.65
GBM 6	0.59	0.23	0.32	4.33
GBM 13	0.34	0.11	0.31	2.84
GBM 8	0.03	0.01	0.03	0.38
**p-value**	**0.0004**	**0.0011**	**0.0379**	**0.0027**

Table S6: Spearman's correlation between FAT1 and TGF-β pathway genes in TCGA GBM cases (n=572)

**Correlations**						
			**FAT1**	**TGF-β1**	**TGF-β2**	**SERPINE1**
Spearman's rho	**FAT1**	Correlation Coefficient	1.000	.119**	.123**	.022
		Sig. (1-tailed)		.002	.002	.298
	**TGF-β1**	Correlation Coefficient	.119**	1.000	.112**	.383**
		Sig. (1-tailed)	.002		.004	.000
	**TGF-β2**	Correlation Coefficient	.123**	.112**	1.000	.150**
		Sig. (1-tailed)	.002	.004		.000
	**SERPINE1**	Correlation Coefficient	.022	.383**	.150**	1.000
		Sig. (1-tailed)	.298	.000	.000	
**Correlation is significant at the 0.01 level (1-tailed).						
*Correlation is significant at the 0.05 level (1-tailed).						

Table S7: Spearman's correlation between FAT1 and TGF-β pathway genes in GLASS glioma cases (n=102)

**Parameter**	**TGF-β1**
**Number of XY Pairs**	102
**Spearman r**	0.3518
**P value (one-tailed)**	0.0001
**P value summary**	***

Table S8: Fold expression values of FAT1, PD-L1, PD-L2 and IL-10 genes in 48 GBM samples calculated with respect to normal brain levels and normalized by 18S rRNA internal control.

GBM ID	FAT1	PD-L1	PD-L2	IL-10
GBM 1	0.64	0.17	0.43	18.55
GBM 2	2.80	1.65	6.02	13.42
GBM 3	4.52	0.21	6.37	10.23
GBM 4	10.63	0.70	2.75	0.00
GBM 5	2.61	0.37	4.31	17.36
GBM 6	0.59	0.29	0.03	6.97
GBM 7	5.35	0.24	6.00	25.92
GBM 8	0.03	0.02	0.16	0.00
GBM 9	7.01	0.54	2.25	20.91
GBM 10	2.07	0.64	3.37	0.00
GBM 11	7.62	0.51	4.88	0.00
GBM 12	2.32	0.09	2.11	59.31
GBM 13	0.34	0.08	0.31	17.33
GBM 14	3.18	0.75	10.60	43.63
GBM 15	3.18	9.02	2.93	11.00
GBM 16	3.74	1.55	7.42	5.10
GBM 17	6.99	0.62	17.65	1.60
GBM 18	17.35	0.66	23.29	67.07
GBM 19	34.13	0.41	6.26	14.33
GBM 20	8.78	0.00	0.26	1.68
GBM 21	41.88	19.72	39.09	23.67
GBM 22	8.84	0.57	1.00	3.08
GBM 23	5.05	0.60	8.16	2.07
GBM 24	7.14	1.20	0.00	2.73
GBM 25	4.71	2.69	12.52	6.59
GBM 26	3.12	0.02	0.19	44.82
GBM 27	7.83	0.03	0.31	4.08
GBM 28	3.91	0.20	3.05	5.88
GBM 29	7.07	0.10	1.40	7.28
GBM 30	2.78	0.18	2.61	9.75
GBM 31	1.56	0.05	0.24	4.44
GBM 32	8.59	0.16	2.11	18.17
GBM 33	9.22	2.57	7.33	29.03
GBM 34	6.83	3.56	4.03	4.68
GBM 35	3.83	4.96	99.75	56.26
GBM 36	78.96	0.44	4.64	139.97
GBM 37	16.18	N.A.	N.A.	N.A.
GBM 38	14.64	0.05	1.26	24.57
GBM 39	4.40	0.27	10.40	5.58
GBM 40	6.12	1.87	1.40	4.37
GBM 41	6.00	0.52	0.42	1.85
GBM 42	23.00	1.38	2.67	15.04
GBM 43	21.43	0.16	3.84	7.53
GBM 44	6.39	0.23	4.43	2.27
GBM 45	6.23	0.28	4.32	1.69
GBM 46	2.54	0.13	1.42	3.23
GBM 47	13.31	0.51	5.23	13.47
GBM 48	3.59	0.01	0.01	0.00
GBM 49	31.58	0.32	1.01	32.56

Abbreviation: N.A. - Not available
